# Singapore’*s Anopheles sinensis* Form A is susceptible to *Plasmodium vivax* isolates from the western Thailand–Myanmar border

**DOI:** 10.1186/s12936-017-2114-3

**Published:** 2017-11-16

**Authors:** Sook-Cheng Pang, Chiara Andolina, Benoit Malleret, Peter R. Christensen, Sai-Gek Lam-Phua, Muhammad Aliff Bin Abdul Razak, Chee-Seng Chong, Daiqin Li, Cindy S. Chu, Bruce Russell, Laurent Rénia, Lee-Ching Ng, Francois Nosten

**Affiliations:** 1 Environmental Health Institute, National Environment Agency, 11 Biopolis Way, Singapore, 138667 Singapore; 2 Centre for Tropical Medicine and Global Health, Nuffield Department of Medicine Research Building, University of Oxford, Old Road Campus, Oxford, UK; 3 Shoklo Malaria Research Unit, Mahidol-Oxford Tropical Medicine Research Unit, Faculty of Tropical Medicine, Mahidol University, Mae Sot, Thailand; 4 Singapore Immunology Network (SIgN), A*STAR, 8A Biomedical Grove, Singapore, 138648 Singapore; 5 Department of Microbiology and Immunology, University of Otago, 720 Cumberland St, Dunedin, 9016 New Zealand; 6 Department of Microbiology and Immunology, Yong Loo Lin School of Medicine, National University of Singapore, National University Health System, 5 Science Drive 2, Blk MD4, Level 3, Singapore, 117597 Singapore; 7 Department of Biological Sciences, National University of Singapore, 14 Science Drive 4, Singapore, 117543 Singapore

**Keywords:** Malaria vector, Infection, *Anopheles sinensis* Form A, *An. cracens*, Sporozoites

## Abstract

**Background:**

Singapore has been certified malaria-free by the World Health Organization since November 1982. However, sporadic autochthonous malaria outbreaks do occur. In one of the most recent outbreaks of vivax malaria, an entomological investigation identified *Anopheles sinensis* as the most probable vector. As metaphase karyotype studies divided *An. sinensis* into two forms, A and B, with different vector competence: the investigation of vector competence of *An. sinensis* found in Singapore was thus pursued using *Plasmodium vivax* field isolates from the Thailand–Myanmar border.

**Methods:**

Adults and larvae *An. sinensis* were collected from Singapore from 14 different locations, using various trapping and collection methods between September 2013 and January 2016. Molecular identification of *An. sinensis* species were conducted by amplifying the *ITS2* and *CO1* region using PCR. Experimental infections of *An. sinensis* using blood from seven patients infected with *P. vivax* from the Thailand–Myanmar border were conducted with *Anopheles cracens* (*An. dirus* B*)* as control.

**Results:**

Phylogenetic analysis showed that *An. sinensis* (F_22_, F_2_ and collected from outbreak areas) found in Singapore was entirely Form A, and closely related to *An. sinensis* Form A from Thailand. Artificial infection of these Singapore strain *An. sinensis* Form A resulted in the development of oocysts in four experiments, with the number of sporozoites produced by one *An. sinensis* ranging from 4301 to 14,538.

**Conclusions:**

Infection experiments showed that *An. sinensis* Form A from Singapore was susceptible to Thai–Myanmar *P. vivax* strain, suggesting a potential role as a malaria vector in Singapore.

## Background

Singapore was once rampant with malaria cases [1]. Outbreaks in mainland Singapore and off-shore islands of Singapore involving the known malaria vectors, i.e. *Anopheles maculatus*, *Anopheles epiroticus* (previously known as *Anopheles sundaicus*) and *Anopheles letifer*, were reported from 1960s to 1970s [2–4]. Singapore attained its malaria free status in November 1982 [2]. The total malaria annual incidence rate fluctuated between 2.9 and 3.9 cases per 100,000 people from 1998 to 2007, and 0.5 to 2.6 per 100,000 people from 2008 to 2015 [5]. The major causative parasite was *P. vivax*, followed by *P. falciparum*. While almost all cases were imported cases, there have been occasional sporadic malarial cases with no travel history (e.g. in 2010 and 2013) and 15 small sporadic localized transmissions with less than 50 cases in each outbreak [5–9]. As a tourist and business hub, with high reliance on foreign personnel from malaria endemic countries, Singapore remains vulnerable to malaria unless the vector population is well understood and remains well controlled.

The last outbreaks occurred in the middle of 2009, when three clusters with a total of 29 *vivax* malaria patients, with no recent travel history, were identified by the Ministry of Health. Relapse cases in *vivax* malaria amongst foreign workers from malaria endemic countries are common and defining if the cluster is due to local transmission is challenging. Therefore, molecular epidemiology was performed using the *msp3a* and *msp1* genes of the parasite. It confirmed only two independent local transmissions in Mandai-Sungei Kadut and in Sembawang [8]. The predominant *Anopheles* found in the two areas was *Anopheles sinensis*, a mosquito that was not previously recognized as a vector in Singapore. Transmission in Jurong could not be confirmed as the infecting parasite from the cases showed no genetic link among them. Correspondingly, no potential Anopheles vectors, including *An. sinensis*, were found in the vicinity. Although *An. sinensis* has been implicated as the malaria vector in some parts of Asia, including Korea, China, Japan and Vietnam, it has never been reported as a vector in Singapore [10–23].

*Anopheles sinensis* is a member of the *Hyrcanus* group. Due to morphological complexity and similarity among the members of the group, the members have often been misidentified and their respective vector status is confusing [24, 25]. Furthermore, confirming *An. sinensis* as vector has been made more complicated by the existence of two forms, i.e. Form A and B, both of which are morphologically identical [26–28]. Yet, hybridization of these two forms showed they were genetically compatible, yielding viable progeny, complete synaptic polytene chromosomes and was said to exhibit cytological polymorphic races [29, 30].

The vector competence of these two forms of *An. sinensis* is not fully understood. To date, only a single study reported that *An. sinensis* Form B was able to produce sporozoites in the salivary glands, while Form A could not [31]. Based on the cytological polymorphism of *An. sinensis* and on previous vector competence studies [29–31], it was noteworthy that the two forms could have different vector abilities in malaria transmission depending on their geographic regions. This study aims to characterize Singapore’s strain of *An. sinensis*, including its vector competence.

## Methods

### Mosquito collection

**Fig. 1:**
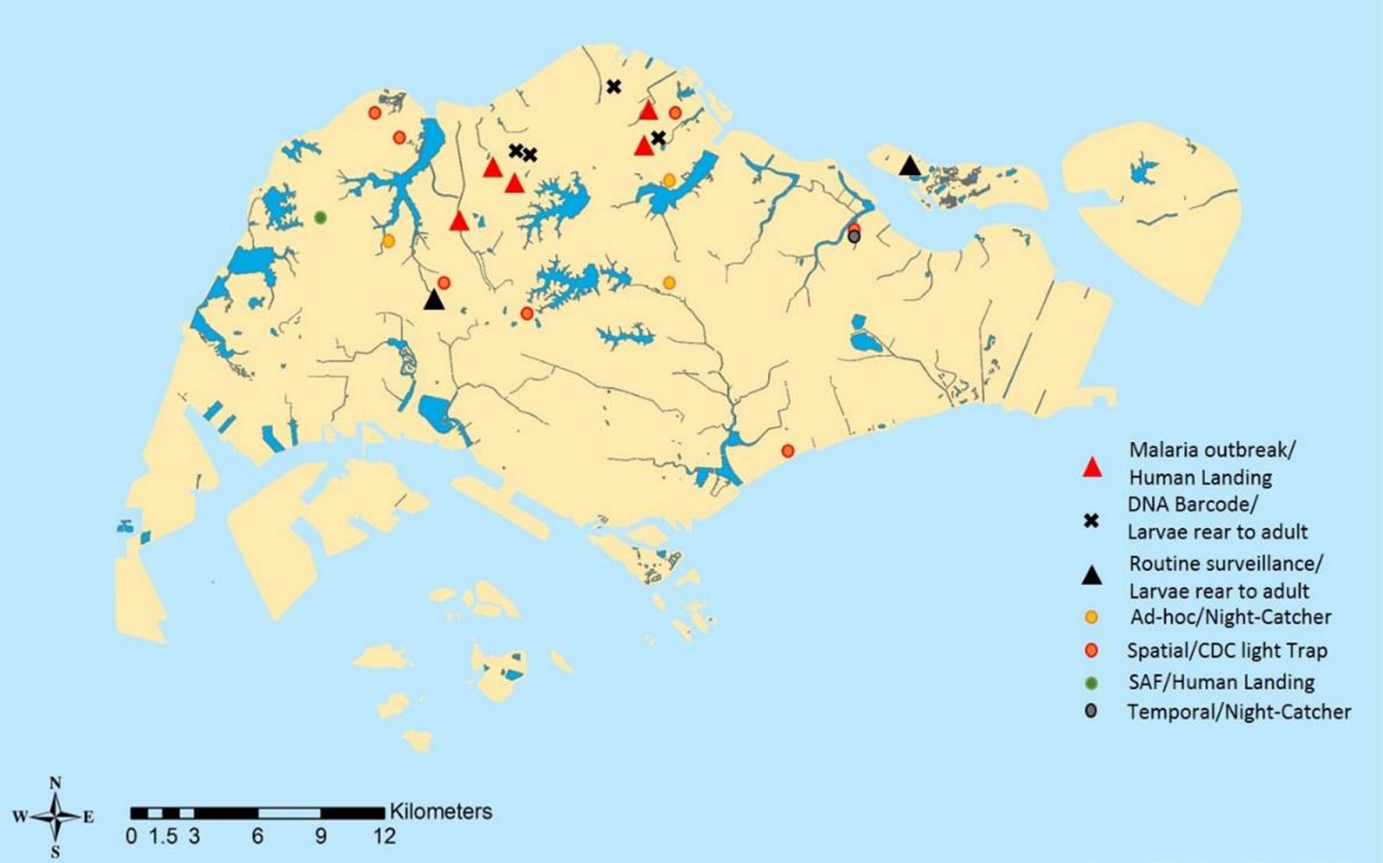
Spatial distribution of *Anopheles sinensis* collected from 13 locations throughout Singapore for determining the taxonomic forms of Singapore’s *An. sinensis*. Additionally, sequences of samples from four locations labelled “x” and five locations labelled as “∆” were extracted from Genbank as references. Note: Spatial/CDC light trap: Spatial distribution study conducted in 2013 using CDC light trap; Singapore Armed Forces/Human Landing: Mosquitoes collected from Singapore Military training grounds via Human Landing method; Ad hoc/Night-Catcher: Mosquitoes collected due to feedback on high Anopheles adult population using Night-Catcher; Temporal/Night-Catcher: *An. sinensis* collected from a 2 years temporal study using Night-Catcher; Routine surveillance/Larvae reared to adult: Larvae collected through routine surveillance were sent into EHI lab for identification and were reared to adult stage; DNA barcode/larvae reared to adult stage: *Anopheles sinensis* larvae were collected for DNA barcode project (DNA sequences were retrieved from Genbank and were used as reference in our current taxonomic study [32]); Malaria outbreaks/Human Landing: Adult *An. sinensis* were collected during 2009 malaria outbreak via Human Landing (DNA sequences were retrieved from Genbank for our current study [8])

In December 2013, larvae of the *An. sinensis* were collected from a grassy pool of a big field at Changi Coast Road, eastern Singapore and they were colonized in the laboratory. Following the first collection, 103 adults and larvae *An. sinensis* were collected from 13 different locations in Singapore between September 2013 and January 2016 (Fig. 1). *Anopheles sinensis* larvae were collected by the Environmental Health Officer (EHO) of National Environment Agency (NEA) during the routine malaria surveillance and were submitted to Environmental Health Institute (EHI) for identification. They were reared to adult for this study.

**Fig. 2:**
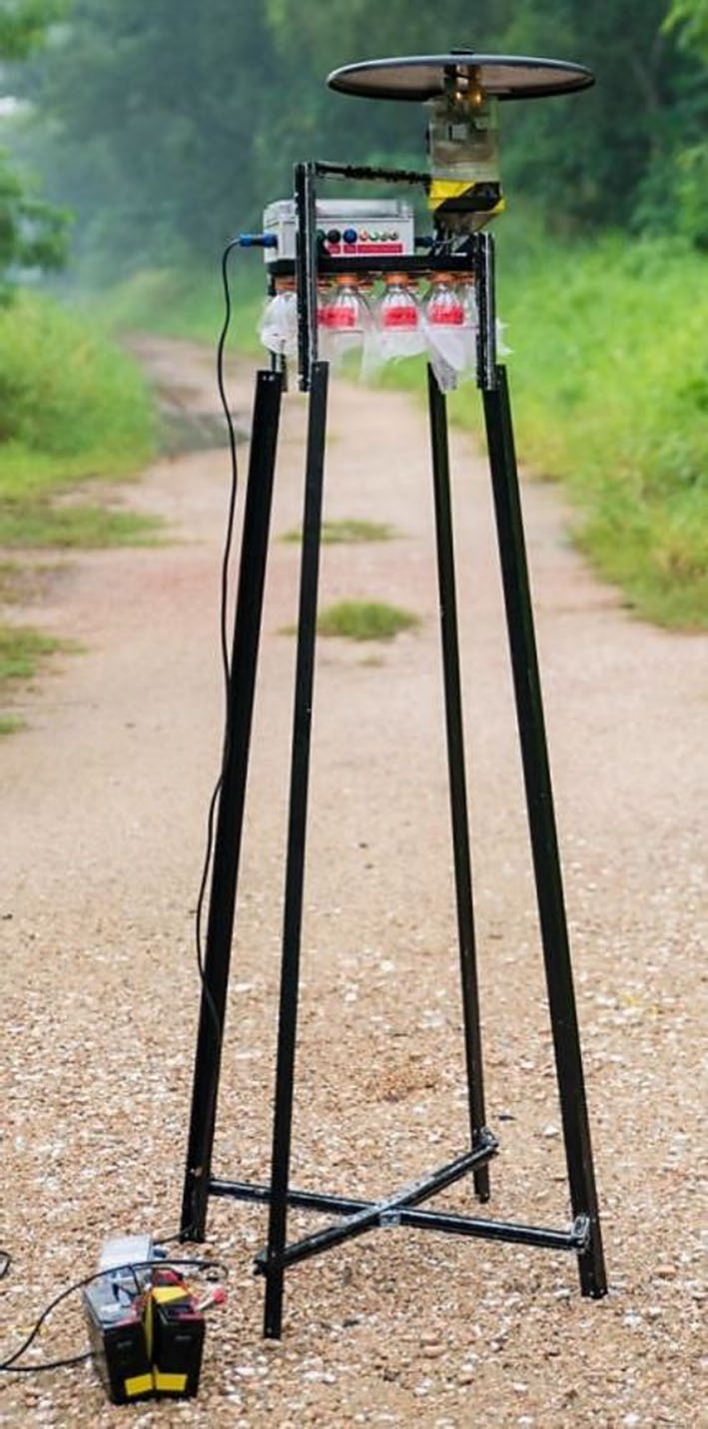
Night Catcher is an in-house designed mosquito trap that enables trapping of mosquitoes at every hour. It was improvised from CDC light trap which uses incandescent light and dry ice (CO_2_) as attractants

For the *An. sinensis* adults, some were collected using modified CDC-light traps during spatial distribution study and ad-hoc collection in response to public feedback on high mosquito population. A portion of adult *An. sinensis* were collected through human landing catch during 2009 malaria outbreak in malaria cluster areas [8] and during surveillance by the Singapore Armed Forces in military training grounds [32]. The remaining adult *An. sinensis* were collected via Night-Catcher during temporal study and ad-hoc collection was conducted due to high mosquito population. Night-Catcher, an in-house mosquito trap, which was improvised from CDC light trap, enables hourly collection of mosquitoes using incandescent light and dry ice (CO_2_) as attractant (Fig. 2). All collections were conducted from 7 p.m. to the 10 a.m. the next morning.

### Mosquito morphological identification

Larvae and adult mosquitoes were identified under compound microscope according to taxonomy keys [17, 33, 34]. Confirmed *An. sinensis* were reared in EHI’s insectary at 25 °C (± 2 °C) and 70% (± 10%) relative humidity. Upon emergence, the adults were then reconfirmed morphologically to the species level according to taxonomy keys [17, 33, 34]. Due to the absence of morphological trait differences between the two forms of *An. sinensis*, these mosquitoes were further determined using molecular taxonomic tools to ensure the accurate form determination as well as purity of the colony.

### Molecular taxonomy

In order to identify the forms of *An. sinensis* in Singapore, regions of both the *COI* and rDNA internal transcribed spacer (*ITS2*) genes were sequenced. All 103 *An. sinensis* collected from 13 different locations were individually processed. Total DNA were extracted individually using DNeasy blood and tissue kit following the manufacturer’s procedures (Qiagen, Hilden, Germany) and stored at − 20 °C until analysis. Two regions flanking the mitochondrial *COI* gene and *ITS2* gene were amplified by polymerase chain reaction (PCR) as described in previous studies [35–37]. Amplicons were then visualized on 2% agarose gel stained with GelRed (Biotium Inc., USA), and cleaned using Purelink PCR purification kit (Invitrogen Corp., USA) according to manufacturer’s instructions. Sequencing was carried out by a commercial laboratory using BigDye Terminator Cycle Sequencing kit (Applied Biosystems, USA). For the susceptible study, sixty adult mosquitoes that were used in the susceptible study were transferred individually into separate 2 ml vials and homogenized using a mixer mill (Retsch Mixer Mill MM301). The DNA extraction, PCR and gel visualization protocols were similar to what was described above. The sequences of the remaining 11 samples from nine different locations were extracted from EHI’s previously published data [8, 35].

### Phylogenetic analysis and genetic distance calculation

Contiguous sequences of *CO1* and *ITS2* genes were created using Lasergene 9.0 software suite (DNASTAR Inc., USA). These sequences were then aligned using Clustal W algorithm [38] executed in BioEdit *v7.05* software [39]. Neighbour joining algorithm was adopted during the construction of phylogenetic trees using MEGA 6.06 software suite [40]. Parameters selection included a Kimura-2 parameter substitution model with gamma distributed rates using the nearest neighbour interchange heuristic search method. Robustness of clustering was determined by bootstrap analysis with 1000 replicates. Reference DNA sequences were obtained from the GenBank database. The pairwise distances between each specimens was computed using MEGA 6.06 software package [40].

### Colonization technique

Colonization was initiated by transferring wild-caught *An. sinensis* adults into 30 cm × 30 cm × 30 cm large cage made from acrylic plastic sheets. Ten percent sucrose was given as food source by soaking it with cotton wool in a glass bottle. Artificial insemination was conducted in earlier generations to propagate and establish a colony in the insectary. Subsequent generations were based on natural insemination, which was induced by exposing these mosquitoes to stroboscopic blue light from 7 p.m. to 9 p.m. for at least 5 days prior to blood feeding [41, 42]. Six days after emergence, females were transferred into 15 oz. transparent plastic container with plastic net affixed on top. Female mosquitoes were deprived from sugar overnight in these feeding containers with moistened cotton pad on top of the net. Specific Pathogen Free (SPF) mini-pig blood was offered to the mosquitoes via a Hemotek^®^ feeding system. On the 3rd day post blood feeding, females were transferred into 15 oz. ovipots (transparent plastic containers) lined with moist filter paper for eggs collection. Eggs were collected on filter paper and hatched in Reversed Osmosis water (RO). Larvae food consisting of wheat germ, oats, dry yeast, casein or low fat milk powder, bubble rice, Vitamin B complex and Nestum were mixed and ground into fine powder. Approximately 0.1 g of larvae food was dispensed daily when the larvae grew from 1st to 2nd instars. Larvae food increased to 0.2 and 0.4 g during 3rd instars and 4th instars, respectively. The second generation (F_2_) of *An. sinensis*, collected from a country club in Singapore in July 2015, was also used in this study to compare the differential vector competence with the lab-bred (F_22_) strain. In order to produce sufficient mosquitoes for the comparison, artificial insemination of F_0_ and F_1_ *An. sinensis* were carried out. This strain was colonized following the above described protocol.

### Transportation of *Anopheles sinensis* eggs

A colony of the twenty-second generation of *An. sinensis* (F_22_) and another of F_2_ were used for the competence study. An approval and an export permit were obtained from Director-General Public Health of National Environment Agency prior to sending the eggs of *An. sinensis* to SMRU. Eggs produced at the insectary of EHI, were transferred onto a clean piece of filter paper, packed and sealed in a sterile petri dish before transporting to Shoklo Malaria Research Unit (SMRU) laboratory, on the Thai-Myanmar border. Although *An. sinensis* Form A and B have previously been found in northern Thailand [28], every precaution was taken to ensure that Singapore’s strain *An. sinensis* used in this study were not released.

### Preparation of patient blood for infection

Patients seeking consultation at SMRU migrant clinics located along the border (Wang Pha, Mawker Thai) where they were tested by blood smear microscopy, only gametocytes positive patients were selected for the study. After a written informed consent was obtained, five to 10 ml of venous blood were drawn into a heparin tube, and immediately placed in a water bath at 37–38 °C to prevent exflagellation of male microgametes [43]. Within an hour, blood samples were transported from the field clinics to the central SMRU laboratory for processing. Following centrifugation at 1800*g* for 5 min in an Eppendorf^®^ centrifuge which was warmed at 38 °C, plasma was replaced with AB+ serum and within 10 min the blood was transported to the insectary.

### Mosquito infection in secure insectary at Maesot

All experimental mosquito infections were carried out at the SMRU secured insectary in Mae Sot as described by Andolina et al. [44]. The secure insectary that is physically separated from open areas by four sealed and locked doors. Only authorized trained personnel can gain access and conduct infection studies. All infected/engorged mosquitoes were counted and placed in incubators (Sanyo^®^, MIR-254) were secured with netting material. Mosquitoes which fed insufficiently were killed in ethanol 70%. *Anopheles cracens* (*An. dirus* B*)*, an efficient *P. vivax* vector [44] was used as a positive control and was fed with the same blood samples, in parallel with *An. sinensis.*

### Microscopy detection of oocysts and sporozoites in mosquitoes

On seven to 8 days post infection, midguts of both mosquito species were dissected and stained with 1% mercurochrome. Oocyst positive midguts were placed in 100 µl of PBS and stored at − 80 °C until PCR was performed. Dissection of salivary glands for sporozoites detection was carried out 15 days post infection. Salivary glands were placed in an Eppendorf tube filled with 50 µl of Roswell Park Memorial Institute medium (RPMI) and kept on ice until the dissection of all mosquitoes was completed. The sample was spun down for 5 min in a micro centrifuge and salivary glands were pooled and crushed using a 100 µl pipette. 10 µl of salivary glands suspension was placed into a KOVA Glasstic slide with 10 grids. Sporozoites were counted and averaged on four grids, multiplied to the chamber factor and dilution factor in order to calculate the number of sporozoites per µl. Average sporozoites counts in a single mosquito was calculated by dividing the total sporozoites with the number of mosquitoes dissected.

### Molecular detection of *P. vivax* in mosquitoes

To confirm *P. vivax* infection, DNA from dissected midguts was extracted in 100 µl PBS using a Qiagen Tissue Kit with minor modifications. Briefly, 180 µl of ATL buffer and 50 µl of proteinase K (Qiagen Tissue Kit) was added to the sample, mixed briefly by vortex and incubated overnight at 56 °C in a shaking incubator. Following digestion, DNA was bound to the silica membrane, washed then eluted in 200 µl water following manufacturer’s instructions. The sample was then concentrated by drying in a vacuum concentrator at 30 °C and re-eluted in 10 µl AE Buffer (Qiagen). Primers and probes described by Perandin et al. [45] were used to amplify and detect species specific regions of the 18S rRNA gene. Real-time PCR was done using QuantiTect Multiplex RT-PCR Kit and an ABI 7500 Fast Cycler.

### Statistical analysis

R-3.1.1 software was used to conduct statistical analysis in this study [46]. Two-way Wilcoxon rank sum test was used when comparison of oocysts development was made between F_2_ and F_22_ *An. sinensis* Form A.

### Ethics approval

The study was approved by Oxford Tropical Research Ethics Committee (Reference 28-09).

## Results

### Taxonomic status of Singapore strain *Anopheles sinensis*

**Fig. 3:**
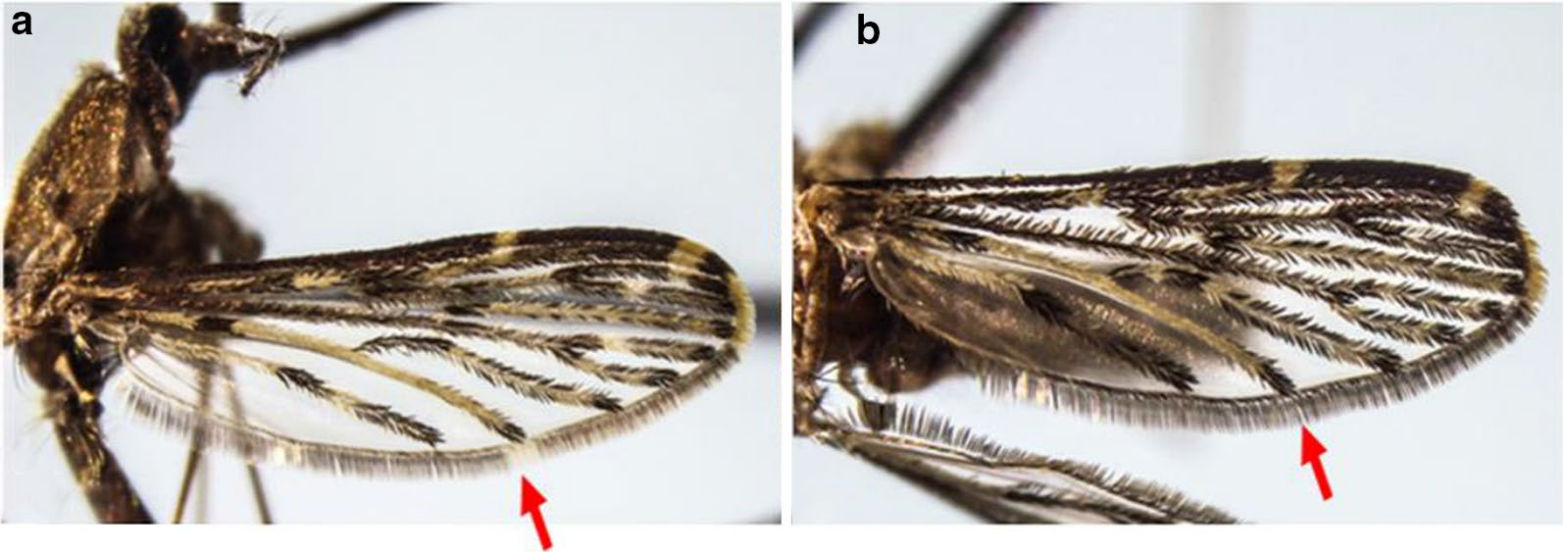
*Anopheles sinensis*’ wing vein CuA (indicated by red arrow) showed **a** pale fringe spot and **b** dark fringe spot [35]

A total of 103 mosquitoes collected from the various locations were confirmed to be *An. sinensis* through morphological identification. However, polymorphic wing variation at CuA was noted among the specimens from field collection (Fig. 3). Out of the 103 specimens, only 42 specimens had complete morphological characteristics where wing scales were still intact. Of the 42 specimens, 20 (47.6%) of them had pale CuA fringe spots, while the remaining 22 (52.4%) showed dark fringe spots. The locality and the proportion of the dark and pale CuA fringe spot are listed in Table 1. It was observed that each location could have both *An. sinensis* with pale and dark CuA fringe spots.

**Table 1:** The proportion of *Anopheles sinensis* with pale or dark CuA wing fringe spot and their respective locations

Location	Pale CuA	Dark CuA
Bishan-AMK Park	11	11
Orchid Country Club	2	1
Lorong Halus	2	2
Lim Chu Kang	4	4
Western Training Plot	0	1
Lorong Semangka	0	2
Bukit Batok Rd	1	2
Total (%)	20 (47.6%)	22 (52.4%)

**Fig. 4:**
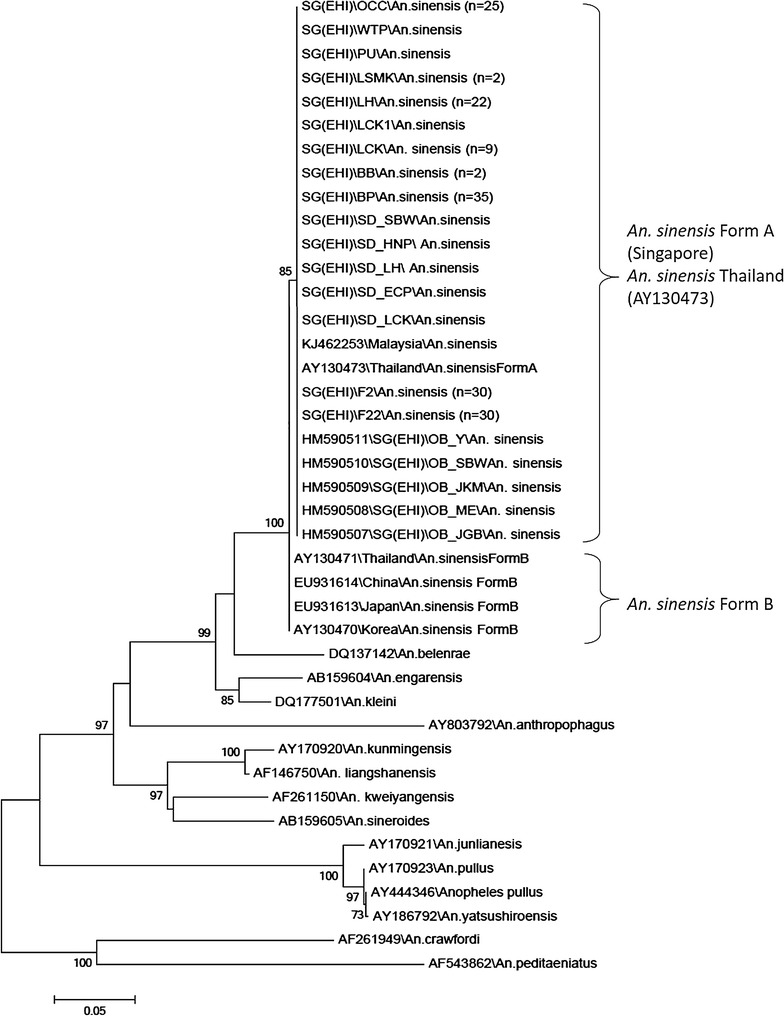
Phylogenetic tree of the *ITS2* genes of *Anopheles hyrcanus* group, constructed using the neighbor-joining algorithm. The values next to the nodes are bootstrap percentages based on 1000 replicates, and only bootstrap percentages above 70% are shown

Phylogenetic analysis based on *ITS2* gene of 103 Singapore *An. sinensis* and 42 reference sequences from the NCBI database showed that all Singapore *An. sinensis* sequences, including those collected during 2009 outbreak, formed a monophyletic clade. Though the Singapore sequences were derived from mosquitoes with CuA pale or dark fringe spots, it is interesting to note the tight clustering despite the differences in wing fringe coloration. The phylogenetic tree also shows that the Singapore *An. sinensis* clustered together with Thailand’s *An. sinensis* Form A (bootstrap value = 85) (Fig. 4). On the other hand, *An. sinensis* Form B from Thailand (AY13047.1), Korea (AY130469.1), China (EU 931614) and Japan (EU 931613) formed a separate clade from our local *An. sinensis* with strong bootstrap support (bootstrap value = 100). None of the Singapore *An. sinensis* adults falls into the Form B clade. The data suggests that *An. sinensis* found in Singapore are Form A.

**Fig. 5:**
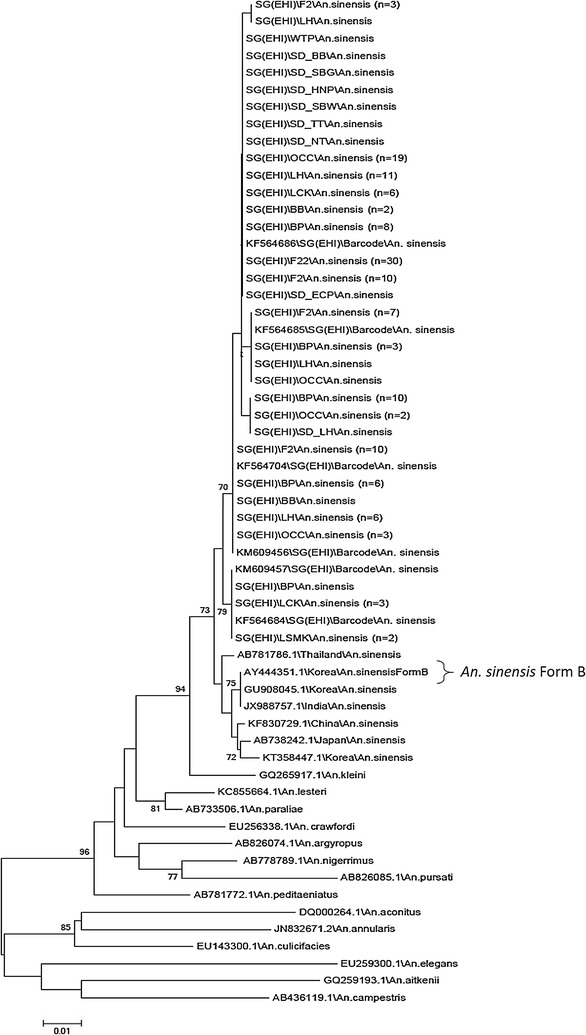
Phylogenetic tree of the *CO1* genes of *Anopheles hyrcanus* group, constructed using the neighbor-joining algorithm. The values next to the nodes are bootstrap percentages based on 1000 replicates, and only bootstrap percentages above 70% are shown

Similarly, the phylogenetic analysis of *CO1* gene sequences using neighbor joining showed that Singapore *An. sinensis* clustered separately from *An. sinensis* Form B from Korea (AY444351) with bootstrap value of 73 (Fig. 5). There was no reference sequence of Form A *CO1* gene in the NCBI database for comparison with Singapore *An. sinensis* sequences. Interestingly, unlike the *ITS2* sequences, the *COI* gene sequences of local *An. sinensis* formed subgroups, though with weak bootstrap support, indicating subtle genetic changes at mitochondrial level.

Previously, it was reported that approximately 75% of *An. sinensis* in the Malaya region (including Singapore) and Borneo had dark fringe spot [34]. However, in this current study, it was interesting to note that 22 (52.4%) of the examined local *An. sinensis* has dark fringe spot at CuA (Table 1). When all 42 specimens with pale and dark CuA wing fringe spots were tallied with the *CO1* phylogenetic tree, no specific clustering of pale or dark phenotype in subgroups. Both phenotypes randomly occurred within the *CO1* phylogenetic tree.

To ensure that the laboratory mosquitoes used for infection is consistent with field caught mosquitoes, the *CO1* and *ITS2* genes of 30 F_22_ mosquitoes were also analysed. They were found to be the same as field caught ones (Figs. 4, 5).

### Oocysts and sporozoites detection in *An. sinensis*

**Fig. 6:**
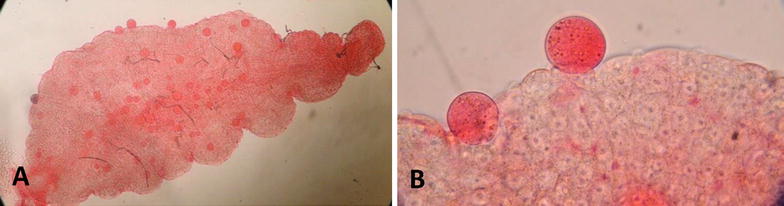
Photos of midguts of *An. sinensis* with growth of *P. vivax* oocysts (red globules). Visualizing midguts with oocysts growth at **a** ×4 magnification and **b** ×10 magnification

The bloods of seven patients with *P. vivax* were fed to F_22_ *An. sinensis* and *An. cracens* using Hemotek^®^ feeding system membrane feeding. In total, 50–100% (Fig. 6) of dissected *An. sinensis* developed one to 92 oocysts (Table 2). Similar results were obtained with *An. cracens* where the infection rate was between 77.7 and 100%, with each mosquito developing one to 200 oocysts. These findings were further confirmed with real-time PCR.

**Table 2:** Detection of *P. vivax* oocysts in midguts and sporozoites in salivary glands of *An. sinensis* and *An. cracens* (control) on 6 Days Post Infection (DPI) and 15 DPI, respectively

Gametocytaemia (gams/500WBC)	Mosquito species	No. of mosquitoes blood fed	Oocysts			Sporozoites	
			Number of mosquitoes with oocysts/dissected (%)	Average number of oocysts (SD)	Range of oocyst densities in each infected mosquito	Average no. of sporozoites in each mosquito	No. of mosquitoes dissected
416	*An. sinensis* (F_22_)	17	9/14 (64.3)	2.6 (± 2.7)	1–8	ND	ND
	*An. cracens*	17	14/17 (82.4)	6.8 (± 6.2)	1–18	ND	ND
480	*An. sinensis* (F_22_)	10	7/7 (100.0)	57 (± 17.4)	27–82	ND	ND
	*An. cracens*	10	7/9 (77.8)	71 (± 42.6)	60–100	ND	ND
576	*An. sinensis* (F_22_)	10	5/5 (100.0)	16 (± 7.7)	3–22	ND	ND
	*An. cracens*	10	5/5 (100.0)	66 (± 26.2)	12–90	ND	ND
384	*An. sinensis* (F_22_)	24	5/7 * (71.4)	6.4 (± 9.1)	2–26	4435	7
	*An. cracens*	20	7/7 (100.0)	8.1 (± 19.9)	2–18	9000	7
768	*An. sinensis* (F_22_)	22	3/3 (100.0)	7 (± 5.3)	3–13	703	8
	*An. cracens*	38	3/3 (100.0)	26 (± 19.3)	9–47	2812	7
416	*An. sinensis* (F_22_)	48	3/3 (100.0)	74 (± 21.6)	50–92	4302	34
	*An. cracens*	47	3/3 (100.0)	>200 (*NA*)	> 200	76,764	34
304	*An. sinensis* (F_22_)	30	2/4 (50.0)	18.8 (± 22.0)	33–42	14,538	24
	*An. cracens*	66	3/3 (100.0)	18.7 (± 3.1)	26–32	4687	24

Data labelled (*) indicated the dissection performed on 15 DPI. ND indicated that dissection for sporozoites was not done

*NA* not available

**Fig. 7:**
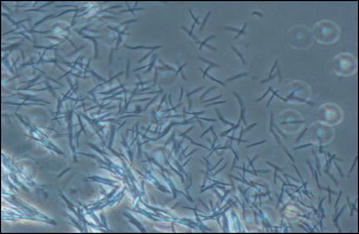
Microscopy image of sporozoites released from salivary glands of infected *An. sinensis* Form A (black arrows). Visualizing sporozoites on KOVA glass slides at ×40 magnification

Out of seven experiments, only the last four yielded enough blood fed mosquitoes for detection of parasite in salivary glands. Salivary glands from each experiment were pooled to ensure minimal loss of sporozoites during manipulation of examining. These four experiments showed that *An. sinensis* could produce 703–14,538 sporozoites per mosquito (Fig. 7), while *An. cracens*, produced 2812 sporozoites to 76,764 sporozoites per mosquito (Table 2).

### Comparison between F_22_ and F_2_ mosquito strains

Comparison of infectivity between F_22_ and F_2_ strains of *An. sinensis* was conducted to determine if they were analogous. Both strains showed similar susceptibility to *P. vivax* infection (two-way Wilcoxon rank sum test, *p* = 0.935) with the F_2_ strain having 75–100% infection rate and F_22_ strain having a 50–100% at midguts (Table 3). Second generation of *An. sinensis* F_2_, had on average 25 and 51 oocysts in each midgut, while F_22_ had 11 and 74 oocysts on average.

**Table 3:** Comparison of oocyst development between F_2_ strain and F_22_ *Anopheles sinensis* Form A

Gametocytaemia (gams/500WBC)	Strains	Oocysts			Sporozoites	
		Positive/dissected (%)	Average no. of oocyst (± SD)	Oocyst range	Number of mosquitoes dissected	Average sporozoites per mosquito
416	F_22_	3/3 (100.0)	74 (± 21.6)	50–80	34	4302
	F_2_	3/3 (100.0)	51.0 (± 9.0)	50–60	35	10,928
304	F_22_	2/4 (50.0)	11 (± 19.1)	33–42	24	14,538
	F_2_	3/4 (75.0)	25 (± 22.1)	33–42	10	11,250

Two-way Wilcoxon rank sum test showed no significant difference in the number of oocysts detected between F_2_ and F_22_ strains (*p* = 0.935). No statistical test was carried out on sporozoites since insufficient data was available.

The average numbers of sporozoites produced by F_22_ were 4302 and 14,538 sporozoites in each mosquito, while that produced by F_2_ strain were 10,928–11,250 sporozoites each. Statistical tests were not carried out on the average number of sporozoites produced due to insufficient data.

## Discussion

In the 2009 malaria outbreaks in Singapore, *An. sinensis*, was the predominant *Anopheles* species found in local outbreak areas. Together with classical and molecular epidemiological data, it was suggested that *An. sinensis* was the probable malaria vector [8]. This study has now determined that *An. sinensis* in Singapore belongs to Form A of the species and more importantly, provided evidence that it is a potential malaria vector in Singapore. Due to limitation in transferring of *Anopheles* eggs and variation of rearing conditions, dissection of few mosquitoes in the initial four experiments could only be carried so as to confirm the successful development of *vivax* oocysts in *An. sinensis* Form A. Following that, minimal number of dissection for oocysts was needed, and infected *An. sinensis* Form A mosquitoes could be reserved for salivary glands dissection on 16 DPI, which is essentially crucial to determine the vector status of *An. sinensis* Form A.

*Anopheles sinensis* is classified under the *Hyrcanus* group. Under this group, it comprises of several species having minute differences in their morphology. From eight species [25], the total number of species within the *Hyrcanus* group increased to 27 [47, 48]. Using integrative taxonomy (the combination of morphological and molecular tools), Singapore’s *An. sinensis* was, for the first time, confirmed to be Form A. All *An. sinensis* collected from the field, including those collected from the 2009 local malaria outbreak [8] formed a clade with Form A of Thailand. No Form B was found. Although *ITS2* showed homogeneity among the *An. sinensis* in Singapore, the *COI* analysis suggest some heterogeneity which probably could only be deciphered using techniques that provides better resolution e.g. Restriction-site Associated DNA sequencing (RADseq) [49] or whole genome sequencing [50].

Although there have been multiple reports of experimental infection that resulted in *An. sinensis* producing sporozoites [51–53], only two [18, 54] natural infections of *An. sinensis* have been recorded in the Southeast Asia. However, these findings were called into doubt [25]. Thailand has never implicated *An. sinensis* as an important malaria vector, with contrasting results in vector competencies being reported from two studies. One reported 61.5% of infected mosquitoes presenting with sporozoites; another showed only 5.88% in Form B and none in Form A [31, 52]. On the contrary, we have shown that Singapore’s strain of *An. sinensis* (Form A) is a potential vector of *P. vivax*, with competency level nearly equivalent to *An. cracens*. It could have been the vector of the 2009 local malaria outbreak. Together with the data from Korea, China and Thailand, the vector competencies of *An. sinensis* appears to be highly dependent on the taxonomic forms [31] and geographical areas of the mosquitoes, and the perhaps genetic diversity parasites [51], Difference due to experimental design also cannot be excluded. More work is needed to understand *An. sinensis* and its role in malaria transmission.

Although we are aware that experimental susceptibility tests do not necessarily reflect the role of malaria transmission in nature, such findings highlight the potential risk of *An. sinensis* if its population is left uncurbed. The habitats of *An. sinensis* in Singapore are not restricted to the rural, usually coastal, areas of Singapore, where typical malaria vectors were found. They appear to thrive well in urban freshwater bodies such as ponds and reservoirs that have become very integrated into the Singapore landscape. Being zoophilic, numerous reports classified *An. sinensis* as an inefficient or an unimportant vector even though infections were detected naturally and experimentally [13, 14, 51]. However, in an urbanized city like Singapore, where animals are scarce, *An. sinensis* could readily bite human since human density is considerably high [13, 15]. Thus, the risk of malaria transmission by *An. sinensis* could not be disregarded, and warrants monitoring and surveillance. During the investigation and mitigation of *An. sinensis* breeding, it was found that they can be controlled by removing algae that develop in these water bodies. More work is ongoing to determine the risk of *An. sinensis* breeding in urban Singapore.

## Conclusions

Together, the data suggests that *An. sinensis* Form A could have been the vector of the 2009 local malaria outbreak and highlights a potential risk of malaria transmission in Singapore by *An. sinensis*. The local map of malaria receptive area for *Anopheles* surveillance and control has been reviewed to include the presence of *An. sinensis*.
